# Founding of the culture collection of antibiotic-resistant strains of zoonotic bacteria in the Russian Federation

**DOI:** 10.14202/vetworld.2023.1451-1460

**Published:** 2023-07-19

**Authors:** Olga Ivanova, Dmitry Blumenkrants, Ekaterina Krylova, Irina Soltynskaya, Anastasia Goncharova, Evgeny Chaikin, Anna Akhmetzyanova, Alexander Panin

**Affiliations:** Federal State Budgetary Institution, The Russian State Center for Animal Feed and Drug Standardization and Quality, Moscow, Russia

**Keywords:** antibiotics, bioresource centers, genes, microorganisms, prevalence, resistance

## Abstract

**Background and Aim:**

The main purpose of a national bioresource center is to standardize, centralize, preserve, and ensure accessibility of microbial bioresources that accumulate there because of state research programs. The establishment of national bioresource centers for antibiotic-resistant microorganisms allows to solve practical problems in the field of veterinary service, as well as to develop effective chemotherapeutic and disinfectant drugs to overcome the mechanisms of resistance. This study aimed to outline the process of forming a national culture collection of antibiotic-resistant strains of zoonotic bacteria in the Russian Federation using two microbial strains.

**Materials and Methods:**

The object of research was isolates of *Salmonella* spp., *Escherichia coli*, *Enterococcus* spp., *Campylobacter* spp., *Listeria monocytogenes*, and *Staphylococcus* spp., all of which were obtained from biomaterials of farm animals, feed samples, bedding, water from livestock buildings, washouts from environmental objects, and food products. The resistance of bacterial isolates was determined using microbiological and molecular-genetic research methods.

**Results:**

During monitoring studies, 1489 bacterial isolates were isolated. In total, 408 bacterial isolates were tested for sensitivity to antimicrobial agents, including *E. coli* (47.6%), *Salmonella* spp. (30.4%), *Enterococcus* spp. (11.3%), and *Campylobacter* spp. (10.8%). For genetic characterization, 95 isolates of *Salmonella enterica*, *E. coli*, *Campylobacter* spp., *L. monocytogenes*, *Staphylococcus* spp., *Enterococcus* spp. were chosen from the research collection, which was formed as part of the monitoring program for antibiotic resistance.

**Conclusion:**

Deposited isolates that underwent whole-genome analysis can be used as positive control samples both in the development and use of methods or test systems for the detection of various resistance genes in zoonotic bacteria. In addition, such isolates can also be used for microbiological studies related to determining the sensitivity of microorganisms to antibacterial drugs, for phenotypic studies in the diagnosis of various bacterial infections in animals and birds, and retrospective analysis of strains from numerous collections.

## Introduction

The main purpose of a national bioresource center is to standardize, centralize, preserve, and ensure the accessibility of microbial bioresources that accumulate there because of state research programs. Its activity plays an important role in ensuring biosafety and technological independence of the state and is also one of the main elements in the structure of harmonization of the quality control system for raw materials and final products [1]. As part of monitoring studies of bacterial resistance to antibacterial drugs for veterinary use, isolates with genes encoding specific proteins that provide various resistance mechanisms, as well as mutations in genes encoding targets for antibiotic action, are of great interest [2]. In accordance with the data of the World Health Organization (WHO), the most relevant studies are on resistant strains of *Salmonella* spp., *Escherichia coli*, *Enterococcus* spp., *Campylobacter* spp., and *Listeria monocytogenes*, *Staphylococcus* spp., which were the causative agents of 5098 outbreaks of food poisonings in the countries of Eurasia in 2021, the mortality rate reached 0.9% [3]. The development of antibiotic-resistant bacteria occurs due to the presence of genes that code specific proteins programmed to destroy antibiotics or protect target of action, or which provide an active efflux of antibiotics. Moreover, the resistance may be linked with the mutations in chromosomal genes that encode targets of action for antibiotics [4–6]. Antibiotic resistance genes are usually associated with the mobile elements of the bacterial genome: plasmids, transposons, integrons, genomic islands, etc. This leads to the possibility of horizontal gene transfer even between taxonomically and ecologically distant microorganisms. Thus, pathogenic microorganisms can obtain resistance genes from the environment. The reverse route is also likely [7–9].

Identifying and studying multidrug-resistant zoonotic bacteria circulating in the agro-industrial complex is one of the priorities of the public health veterinary service, and it emphasizes the importance of the veterinary surveillance system in monitoring the resistance of microorganisms to antibacterial drugs [10, 11]. The priority here is to disclose scientific knowledge in the field of fundamental research of ecological plasticity and adaptation of microorganisms to the effects of antibacterial drugs for establishing national bioresource centers. This will solve applied problems of the veterinary service and help to develop effective chemotherapeutic and disinfectant drugs that overcome resistance mechanisms.

In this regard, this study aimed to discuss the process of formation of a research collection of antibiotic-resistant strains of zoonotic bacteria in the Russian Federation, using the example of two microbial strains, *E. coli* and *Salmonella* *enterica*.

## Materials and Methods

### Ethical approval

This study was conducted using isolated microorganisms and does not require permission from the ethics committee.

### Study period and location

The study was conducted from September 2021 to November 2022 at the Division of Biotechnology of Federal State Budgetary Institution, The Russian State Center for Animal Feed and Drug Standardization and Quality, Moscow, Russia.

### The object of research

The object of research was isolates of *Salmonella* spp., *E. coli*, *Enterococcus* spp., *Campylobacter* spp., *L. monocytogenes*, and *Staphylococcus* spp. obtained from farm animals, including washouts of the mucous membrane of the nasal cavity, rectum, and contents of cloaca and feces. Samples of feed, food products, litter, water from livestock buildings, and washouts from environmental objects were also investigated. All studied samples were taken on the territory of agricultural enterprises and retail trade facilities of the Russian Federation.

According to the recommendations of the European Committee on Antimicrobial Susceptibility Testing (EUCAST) and the Clinical and Laboratory Standards Institute, the following reference strains of microorganisms were used as controls: *Escherichia coli* ATCC 25922, *Campylobacter jejuni* ATCC 33560, and *Enterococcus faecalis* ATCC 29212.

### Microbiological methods

The cultivation of microorganisms was carried out at 35 ± 2–45 ± 2°C for 24–48 h in liquid and solid nutrient media. Microorganisms were identified using matrix-assisted laser desorption/ionization time-of-flight mass spectrometry “Microflex LT” (MALDI-TOF-MS) (Bruker Daltonik Inc., Germany), method of direct application. The results were confirmed by conventional microbiological methods in accordance with the classification system “Bergy’s manual 1984–1989”, as well as using commercial test systems [12]. The ability of microorganisms to form biofilms *in vivo* was determined by conventional methods [13]. Morphometric studies were performed with a representative sampling of an accurate frequency of occurrence of ≥90.0% of the field of view of the “Leica DMRB microscope” (“Leica”, Germany).

The study of the sensitivity of microorganisms to 28 antibacterial drugs of 12 classes (Supplementary Table-S1) was carried out using serial dilution method in sterile 96-well microplates (“Corning”, USA) in accordance with guidelines 4.2.1890-04; EUCAST (Antimicrobial Susceptibility Testing, Disk Diffusion Method); Performance Standards for Antimicrobial Disk and Dilution Susceptibility Tests for Bacteria Isolated From Animals, Approved Standard - Fourth Edition.

### Molecular-genetic methods, utilized software and resources

DNA isolation was carried out using the DNA-sorb-B reagent kits (Federal Scientific Research Institute of Experimental Engineering, Russia) in accordance with the manufacturer’s instructions. The DNA library was prepared using the Nextera XT DNA Sample Preparation Kit (Illumina, Inc., USA) according to the manufacturer’s instructions. Whole genome sequencing was performed on the MiSeq system (Illumina, Inc) according to the standard operating procedure.

For the bioinformatics analysis of whole-genome sequencing data and *de novo* genome assembly, the following tools were used: FastQC 0.11.17 [14], Trimmomatic v.0.36 [15], SPAdes 2.11.1 [16], QUAST 4.6.3 [17], MAUVE v.20150226 [18]. Bacterial species identification and multilocus sequence typing (MLST) were performed through the online service of the Center for Genomic Epidemiology of the Danish University of Technology using KmerFinder server (v.3.0.2) [19] and MLST server (v.2.0.4) [20].

The identification of genetic factors that provide bacterial resistance to various antibiotics was carried out using the ResFinder server [21]. The virulence factor database (VFDB) was used to search for the main virulence factors in bacterial genomes [22]. The search for integrons was conducted using the IntegronFinder v5 software [23] and *Salmonella* pathogenicity islands – SPIFinder online service (v. 2.0) [24].

### Statistical analysis

The results of experimental studies were processed by variational statistics, using the software “Statistical Analysis Software” (“Statistics Solutions”, Clearwater, USA), also considering the criterion of reliability (Student’s t-test) at a confidence level of 95%.

## Results and Discussion

As part of a monitoring program for antibiotic resistance in zoonotic bacteria isolated from biomaterial from farm animals, feed samples, bedding, water from livestock buildings, and washouts from environmental objects, as well as from food samples in the period from 2021 to 2022, 2942 strains of bacteria were identified and deposited; the list of microbial strains in the research collection is presented in Table-1.

**Table-1:** Microbial strains in the research collection in 2021–2022.

S. No.	Microorganisms	Number of isolates
1.	*Enterococcus* spp.	1222
2.	*E. coli*	898
3.	*S. aureus*	302
4.	*Campylobacter*	14
5.	*L. monocytogenes*	374
6.	*Salmonella* spp.	132
	Total	2942

*E. coli=Escherichia coli*, *L. monocytogenes=Listeria monocytogenes, S. aureus=Staphylococcus aureus*

Along with the already mentioned bacteria, the research collection was expanded with bacteria of the genera *Arthrobacter*, *Bacillus*, *Bordetella*, *Brevundimonas*, *Citrobacter*, *Corynebacterium*, *Klebsiella*, *Kocuria*, *Lactobacillus*, *Lactococcus*, *Micrococcus*, *Mycobacterium*, *Proteus*, *Pseudomonas*, *Rhodococcus*, *Staphylococcus*, *Streptococcus*, *Vibrio*, *Saccharomyces*, as well as fungi of the genera *Aureobasidium*, *Aspergillus*, *Chaetomium*, *Cladosporium Penicillium*, *Pseudopithomyces*, *and Fusarium*, all of which are part of the All-Russian State Collection of Microorganisms Strains Used in Veterinary Medicine and Livestock. In total, the collection fund of microorganisms has 187 authenticated and characterized reference strains, the safety of which is ensured by the use of long-term storage methods: Lyophilization and cryopreservation at low temperatures (−70.0°C).

Based on the examples of two microbial isolates, the deposition process is demonstrated. At the first stage, the indication and identification of microorganisms was carried out, and then the phenotypic and genotypic resistance of the pure bacterial culture to antibacterial drugs was studied. Isolates that showed resistance to several antibiotics of different classes were subjected to deposition for several reasons: Conduct quality control studies for nutrient media, drugs for veterinary use, and different agricultural products; to study the growth properties of microorganisms when using various combinations of antibiotics; to use isolates for the diagnosis of pathogens of infectious animal diseases; to use isolates as a reference for quality control studies in veterinary monitoring of antibiotic resistance; and to provide food safety control using microbiological indicators.

### Indication and identification of microorganisms

**Figure-1:**
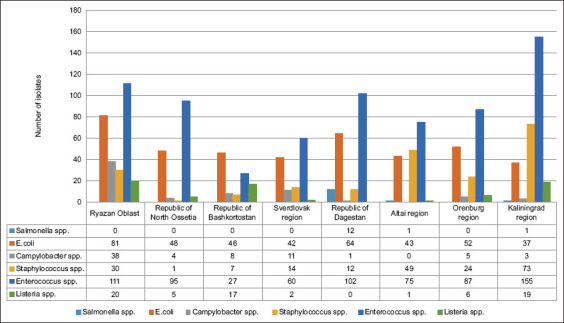
Total number of bacterial isolates from 8 regions of the Russian Federation in 2021–2022.

During monitoring studies, we isolated 1489 bacterial isolates from cows, sheep, pigs, horses, chickens, geese, ducks, and environmental objects (Figure-1). It has been established that the total number of *Campylobacter* isolates significantly exceeded the number of *Salmonella* isolates. The above results confirm the literature data that campylobacteriosis occupies a significant place among acute intestinal infections due to its wide prevalence, the multiplicity of reservoirs, and a trend toward an increase in incidence. Microorganisms of the genus *Campylobacter* as an etiological factor of intestinal infections are more common than *Salmonella*. The significance of the problem of the spread of campylobacteriosis is confirmed by the WHO, that has included this infection in the national program of combating infectious diseases in 108 countries, including the Russian Federation [3].

### Phenotypic resistance of isolates

**Figure-2:**
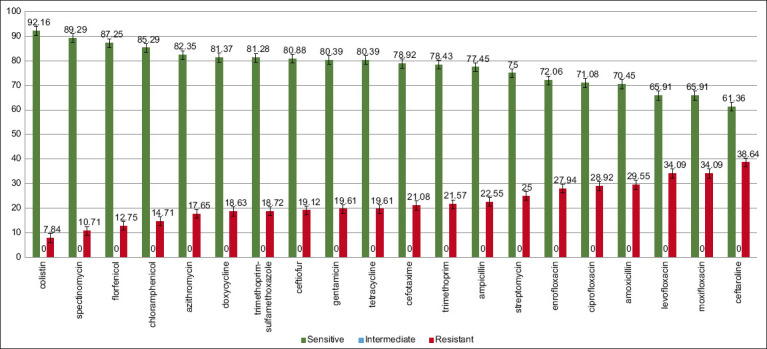
Resistance pattern in *Escherichia coli*.

In total, 408 bacterial isolates were tested for sensitivity to antimicrobial agents, including *E. coli* 47.6%, *Salmonella* spp., 30.4%, *Enterococcus* spp. 11.3%, *Campylobacter* spp. 10.8%. The study of microbiological resistance of isolates was conducted in accordance with the interpretation criteria “EUCAST Epidemiological cut-off values” (ECOFF). When studying the sensitivity of microorganisms by the method of serial dilutions, *E. coli* bacteria were resistant to *β*-lactam group (21.08–38.64%); aminoglycosides (25.0%); and fluoroquinolones (27.94–34.09%) (Figure-2). In samples of biological material from animals at full-cycle food industry enterprises, the vast majority of *E. coli* isolates showed non-lactamase resistance (52.1–64.3%), and the highest prevalence was noted in poultry farms (44.4%), compared with meat enterprises (12.5%), and pig farms (14.3%) [25].

**Figure-3:**
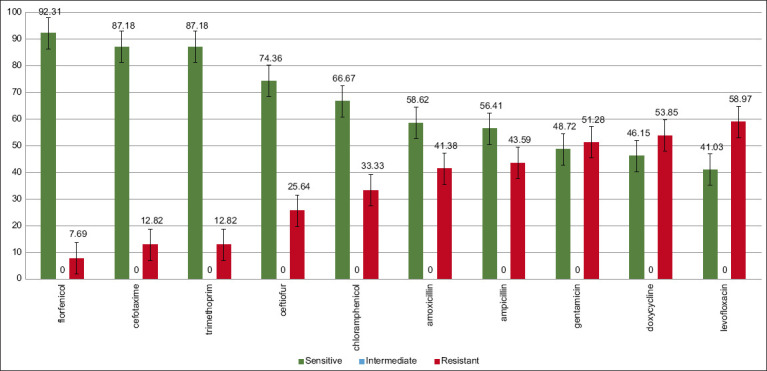
Resistance pattern in *Salmonella* spp.

Bacteria *Salmonella* spp. showed resistance to *β*-lactam group (20.59–41.38%); aminoglycosides (41.38–48.28%), tetracyclines (55.17–65.52%); and fluoroquinolones (68.97%) (Figure-3). Retrospective studies of 2020–2021 indicate a wide distribution (19.6%) of multidrug-resistant serovars of *Salmonella* spp. (19.6%), as well as a high level of resistance to tetracycline (58.0%), sulfonamides (65.0%), ciprofloxacin (46.0%), and gentamicin (42.0%), which is fully consistent with the results of this study [26, 27].

**Figure-4:**
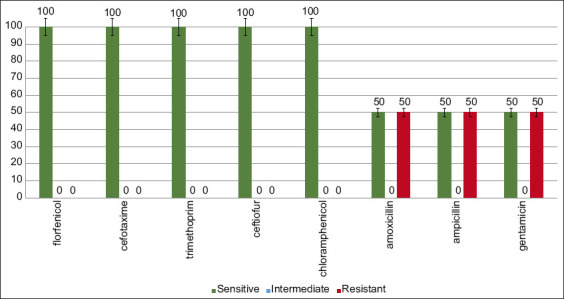
Resistance pattern in *Campylobacter* spp.

The highest indicator of resistance in *Campylobacter* spp. was established in relation to antibiotics of the tetracycline group: tetracycline (50.0%), doxycycline (50.0%), as well as to fluoroquinolones (50.0%) (Figure-4). Fluoroquinolone-resistant *Campylobacter* is in a high-priority group for developing new antibiotics on the WHO list [3].

**Figure-5:**
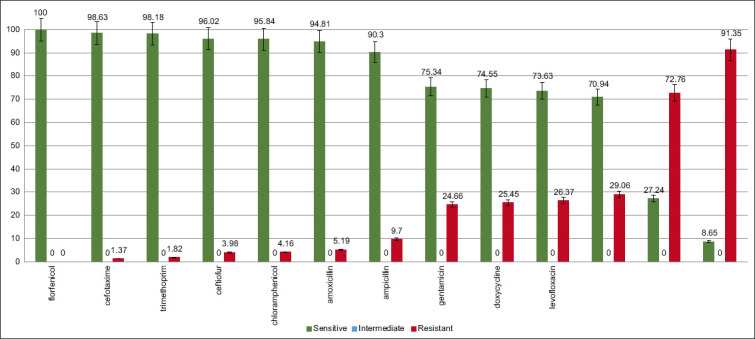
Resistance pattern in *Enterococcus* spp.

Bacteria of *Enterococcus* spp. were resistant to the fluoroquinolones group (24.66%); tetracyclines (25.45–26.73%); macrolide (29.06–72.75%); polypeptides (91.35%) (Figure-5). In the study of the formation of general patterns of a three-dimensional multilayer heterogeneous structure of biofilms *in vitro* by multidrug-resistant isolates, it was found that all the studied microorganisms were strong producers of biofilms, and the optical density (OD) of the sample exceeded the ODs of the control by more than 4 times (ODs = 0.528 ± 0.31). High ODs of biofilms (ODs ≥ 0.400) are linked to multidrug resistance [13]. A direct correlation has been established between the ability of bacteria to form biofilms and the profile of resistance to antibacterial drugs: Multidrug-resistant strains were classified as strong biofilm producers – 91.07% [28].

### Genetic characteristics of isolates of zoonotic bacteria from the research collection of the Federal State Budgetary Institution and The Russian State Center for Animal Feed and Drug Standardization and Quality

For genetic characterization, 95 isolates of *S. enterica*, *Escherichia coli*, *Campylobacter* spp., *L. monocytogenes*, *Staphylococcus* spp., and *Enterococcus* spp. were obtained from the research collection as part of antibiotic resistance monitoring. The selection was carried out on the basis of microbiological data on phenotypic resistance: All 95 isolates were resistant to several antibiotics of various classes. Whole genome sequencing of selected isolates and bioinformatics analysis of the obtained data was then carried out. In addition, 18 were deposited to the All-Russian State Collection of Microorganisms Strains Used in Veterinary Medicine and Livestock.

Genetic characterization aims to evaluate the prevalence of genetic determinants of resistance among zoonotic bacteria isolated from productive animals and food and feed products. The whole-genome sequencing data make it possible to reveal the presence of resistance genes and establish their localization in mobile elements, the structure and features of gene cassettes. The combination of conventional microbiological methods with molecular-genetic methods allows to obtain the most complete information about various characteristics of bacteria, as well as to confirm the phenotypic properties of isolates. In the example of four isolates (Table-2), we present an algorithm for performing genetic characterization and a data’s format for isolates from the research collection of the VGNKI.

**Table-2:** Isolates of *E. coli* and *S. enterica.*

Isolate	Taxonomy, Score^a^	Sample type/source	Year	Region
VGNKI-343	*E. coli*, 2.356 ± 0.09	Feces/turkey	2021	Belgorod region
VGNKI-513	*E. coli*, 2.418 ± 0.17	Vaginal flush/cattle	2021	Kaluga region
VGNKI-2105	*Salmonella* spp., 2.459 ± 0.15	Food/broiler	2021	Kemerovo region
VGNKI-2108	*S.* Infantis, 2.379 ± 0.54	Feces/broilers	2021	Kemerovo region

a Indication of ribosomal bacterial proteins using matrix-assisted laser desorption/ionization time-of-flight mass spectrometry (MALDI-TOF-MS). *S. enterica*=*Salmonella* *enterica*, *S.* Infantis=*Salmonella* Infantis, *E. coli=Escherichia coli*

The quality of sequencing data (FASTQ files) was assessed using the FastQC_0.11.17. Removal of technical sequences and low-quality nucleotides was performed in the Trimmomatic v.0.36. Following parameters were used: NexteraPE-PE.fa: 2:30:10, SLIDINGWINDOW: 4:15. *De novo* assembly of bacterial genomes was performed using the SPAdes 2.11.1 assembler with sequencing error correction and automatic selection of the k-mer length (21, 33, 55, 77, 99). Contigs <500 bp were excluded from further analysis.

An assembly with a combination of the following criteria was chosen as the best: the smallest number of contigs, the largest value of N50, the total length of the contigs and the correct GC content (the proportion of guanine and cytosine bases in the DNA molecule) composition for the analyzed microorganism. The main characteristics of the assembly were obtained using QUAST 4.6.3 and are presented in Table-3.

**Table-3:** Main characteristics of draft genome assemblies*.*

Isolate	Number of contigs	Max length of contig, bp	Total length of contigs, bp	N50	GC content, %
*E. coli* VGNKI-343	99	362591	5106267	152697	50.7
*E. coli* VGNKI-513	86	578888	5353050	188100	50.7
*S.* Bredeney VGNKI-2105	41	661901	4823295	414752	51.8
*S.* Infantis VGNKI-2108	87	599704	5234560	240678	51.8

*S.* Bredeney=*Salmonella* Bredeney, *S.* Infantis=*Salmonella* Infantis, *E. coli=Escherichia coli*, GC content=the proportion of guanine and cytosine bases in the DNA molecule

To determine a bacterial species, we used the search for common k-mers implemented in the KmerFinder. Multilocus typing of the *S. enterica* samples was performed using MLST at loci *aroC*, *dnaN*, *hemD*, *hisD*, *purE*, *sucA*, *thrA*. Typing of the *E. coli* samples at loci *adk*, *fumC*, *gyrB*, *icd*, *mdh*, *purA*, *and recA*. MLST allele sequence and profile data were obtained from PubMLST.org. Genotyping data (Table-4) confirmed the results that were obtained by conventional microbiological methods (tinctorial, morphological, and biochemical).

**Table-4:** Genotyping results.

Isolate	MLST	KmerFinder
*E. coli* VGNKI-343	ST-224	NZ_LR130552 *E. coli* strain MS14386
*E. coli* VGNKI-513	ST-1079	NC_017635 *E. coli* W
*S.* Bredeney VGNKI-2105	ST-897	NZ_CP007533 *S. enterica* subsp. enterica serovar Bredeney strain CFSAN001080
*S.* Infantis VGNKI-2108	ST-32	NZ_CP047881 *S. enterica* subsp. enterica serovar Infantis strain 119944

*S.* Bredeney=*Salmonella* Bredeney, *S.* Infantis=*Salmonella* Infantis, *E. coli=Escherichia coli*, MLST=Multilocus sequence typing

The contigs were ordered using the MAUVE v.20150226 by the nucleotide sequence of the: *Salmonella enterica* strain FSIS1502916 (CP016408.1); *E. coli* strain AR_452 (CP030331.1). The annotation was performed using the RAST server on the open platform SEED for comparative analysis of genomes [29].

Identification of antibiotic resistance genes was carried out by the ABRicate software [30] using BLASTN and BLASTX against nucleotide and amino acid sequences from ResFinder and NCBI BARRGD [31]. The following criteria were used to analyze contig sequences: >95% identity, >80% coverage. The assortment of the identified genes is largely consistent with the multidrug resistance phenotype of isolates (Table-5). Point mutations in DNA-gyrase (*gyrA*) and topoisomerase IV (*parC*, *parE*) genes were found in phenotypically ciprofloxacin- and enrofloxacin-resistant isolates.

**Table-5:** Correlation of AMR phenotypes with AMR genes identified by ResFinder in WGS contigs of *E. coli* and *Salmonella* isolates*.*

Antibiotic	Antibiotic class	*E. coli* 343		*E. coli* 513		*Salmonella* 2105		*Salmonella* 2108	
			
		BMD^a^	NGS^b^	BMD	NGS	BMD	NGS	BMD	NGS
Ampicillin	Beta lactams	R^c^	***TEM-1***	R	***TEM-1, CTX-M-14***	R	***TEM-1, CTX-M-15***	R	***CTX-M-14***
Amoxicillin		R		R		R		R	
Cefotaxime (III generation)		S^d^		R		R		R	
Ceftiofur (Veterinary drug)		S		R		R		R	
Ciprofloxacin	Fluoroquinolones	R	*gyrA_D87N, S83L parC_S80I, parE_S458A*	R	***qnrS1***	R	***qnrB19***	R	*gyrA-p.S83Y, qnrE*
Enrofloxacin (Veterinary drug)		R		R		R		R	
Doxycycline	Tetracyclines	R	*tetB*	R	*tetB*	S		R	***tetA/tetR,*** *tetB*
Streptomycin	Aminoglycosides	R	***aadA1, aadA2, aac (3)-IIa***	R	***aadA1, aadA2, aac (3)-IId***	S		R	***aadA2, strA/strB* *aac (6’)-Ib3***
Gentamicin		R		R		S		S	
Chloramphenicol	Fenicols	R	*cmlA1*	R	*floR, catA1*	S		R	***floR***
Florfenicol (Veterinary drug)		S		R		S		R	
Colistin	Polymyxins	R	***mcr-1***	R		S		S	
Sulfomethoxazole	Sulfonamides	R	***sul3***	R	***sul1***	R		R	***sul1, sul2***
Trimethoprim	Trimethoprim	R	***dfrA14***	R	***dfrA12***	S		R	***dfrA12, dfrA14***

a BMD indicates antimicrobial susceptibility tested by broth microdilution.

b NGS indicates antimicrobial resistance tested by next generation sequencing. ^c^R indicates the isolate resistant or intermediately resistant by at least one criterion (ECOFF or CBP). ^d^S indicates the isolate susceptible by at least one criterion (ECOFF or CBP). Bold font indicates genes located in plasmid contigs. AMR=Antimicrobial Resistance

**Figure-6:**
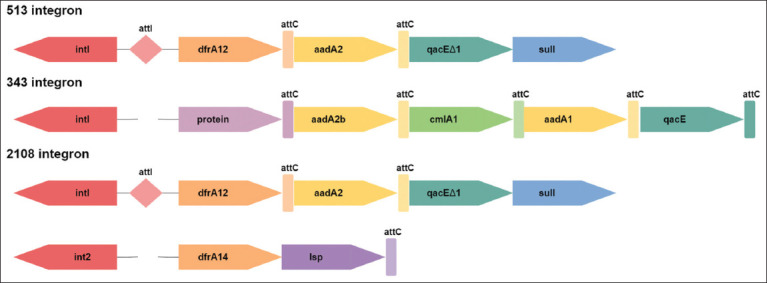
Composition of integrons in *Escherichia coli* and *Salmonella enterica*.

The classification of contigs into chromosomal and plasmid ones was carried out using our own algorithm at Python 2.7 [32]. In isolate *S*. Infantis VGNKI-2108, a pESI-like mega-plasmid was identified that carried genes for resistance to cephalosporins (CTX-M-14), aminoglycosides (*aadA*), sulfonamides (*sul1*), trimetoprim (*dfrA*), and tetracycline (*tetA/R*) [33]. The IntegronFinder v5 was used with default settings to search of integrons in bacterial genomes. Each of them was localized on plasmid contigs. The compositions of Class 1 and 2 integrons are presented in Figure-6.

The search for virulence factors was carried out in the VFDB database using the following criteria: >95% identity and >80% coverage. The results are presented in Table-6. The following criteria were used to search for pathogenicity islands in *Salmonella* spp. using the SPIFinder online service: >95% identity and >60% coverage. The results are presented in Table-7.

**Table-6:** Virulence factors.

Isolate	Virulence factors
*E. coli* VGNKI-343	fimB, C, D, E, F, G, I; yag/ecpW/D, X/C, Y/B, Z/A
*E. coli* VGNKI-513	fimC, D, E, I; fyuA; irp1.2; ompA; yag/ecpW/D, X/C, Y/B, Z/A; ybtA, E, P, Q, T, U
*S.* Bredeney VGNKI-2105	avrA, cdtb, csgA, B, C, D, E, F, G; fimC, D, F, H, I; invA, B, C, E, F, G, H, I, J; mgtB, C; misLorgA, C; pipb, prgH, I, J, K; sicA, P; sip/sspA/A, B/B, C/C; sop/sigb/d, spaO, P, Q, R, S; spi/ssaC/B, ssaD, E, G, H, I, J, L, N, P, Q, R, S, U, V; sscA, BsseA, C, D, E; sspH 1
*S.* Infantis VGNKI-2108	avrA; csgA, B, C, D, E, F, G; fimC, D, F, H, I; invA, B, C, E, F, G, H, I, J; lpfA, B, C, D, E; mgtB, C “mig-14, misLorgA, C; pipB2, B; prgH, I, J, K; ratb, sicA, P; sifb, sinh, sip/sspA/A, B/B, C/C; sipD, slrP, sopA, D2, D; sop/sigb/d, spaO, P, Q, R, S; spi/ssaC/B, sptP, ssaC, D, E, G, H, I, J, K, M, N, O, P, Q, R, S, T, U, V; sscA, B; sseA, B, C, D, E, F, G, J, K1, K2; sspH 2; steB, C; ybtT

*S.* Bredeney=*Salmonella* Bredeney, *S.* Infantis=*Salmonella* Infantis, *E. coli=Escherichia coli*

**Table-7:** *S.* *enterica* pathogenicity islands*.*

Isolate	Salmonella pathogenicity island
*S.* Bredeney VGNKI-2105	SPI 1, SPI 4, SPI 5, SPI 9
*S.* Infantis VGNKI-2108	SPI 1, SPI 4, SPI 5, SPI 9

*S.* Bredeney=*Salmonella* Bredeney, *S.* Infantis=*Salmonella* Infantis, *S.* *enterica=Salmonella* *enterica*

## Conclusion

This study demonstrates the process of establishing a research collection of antibiotic-resistant strains of zoonotic bacteria on the examples of two types of microorganisms. The stages of indication and identification of microorganisms are described, followed by the determination of the phenotypic and genotypic resistance of a pure bacterial culture to antibacterial drugs. The deposited genome-wide characterized isolates can be used as positive control samples both in the development and in the use of methods or test systems to detect various zoonotic bacteria resistance genes. In addition, such isolates can also be used for microbiological studies related to determining the sensitivity of microorganisms to antibacterial drugs and for phenotypic studies in the diagnosis of various bacterial infections in animals and birds. Using well-characterized isolates, a retrospective analysis of strains from different collections becomes also possible: Assessment of antibiotic resistance, pathogenicity, and other properties.

## Authors’ Contributions

AP and OI: Conceptualized and designed the study. DB, EK, and IS: Conducted the experiments. DB and EK: Data analysis and drafted and edited the manuscript. IS, AG, EC, and AA: Data analysis and interpretation. OI: Revised the manuscript. All authors have read, reviewed, and approved the final manuscript.

## Supplementary Material

**Table-S1:** The list of antimicrobial drugs used in broth microdilution test on resistance.

Classes of antibiotics	Antibacterial drugs	Microorganisms
*β*-lactams	Ampicillin	*Salmonella* spp., *E. coli*, *Campylobacter* spp., *L. monocytogenes*, *Enterococcus* spp.
	Cefotaxime	*E. coli*, *Staphylococcus* spp.
	Amoxicillin	*L. monocytogenes*, *Enterococcus* spp.
	Ceftiofur	*Salmonella* spp., *E. coli*
	Cefotaxime	*Salmonella* spp.
	Ceftaroline	*Salmonella* spp.
Aminoglycosides	Gentamicin	*Salmonella* spp., *E. coli*, *L. monocytogenes*, *Enterococcus* spp., *Staphylococcus* spp.
	Streptomycin	*Salmonella* spp., *E. coli*, *Campylobacter* spp., *Enterococcus* spp., *Staphylococcus* spp.
	Spectinomycin	*E. coli*, *Staphylococcus* spp.
Tetracyclines	Doxycycline	*Salmonella* spp., *E. coli*, *Campylobacter* spp., *Enterococcus* spp., *Staphylococcus* spp.
	Tetracycline	*Salmonella* spp., *E. coli*, *Campylobacter* spp., *Enterococcus* spp., *Staphylococcus* spp.
Macrolides	Erythromycin	*Campylobacter* spp., *L. monocytogenes*, *Enterococcus* spp., *Staphylococcus* spp.
	Virginiamycin	*Enterococcus* spp.
Lincosamides	Clindamycin	*Campylobacter* spp., *Staphylococcus* spp.
Glycopeptides	Vancomycin	*L. monocytogenes*, *Enterococcus* spp., *Staphylococcus* spp.
Polypeptides	Bacitracin	*Enterococcus* spp.
Phenicols	Florfenicol	*Salmonella* spp., *E. coli*, *Enterococcus* spp.
	Chloramphenicol	*Salmonella* spp., *E. coli*, *Campylobacter* spp., *L. monocytogenes*, *Enterococcus* spp., *Staphylococcus* spp.
Polymyxins	Colistin	*Salmonella* spp., *E. coli*,
Rifampicins	Rifampicin	*E. coli*, *Enterococcus* spp., *Staphylococcus* spp.
Diaminopyrimidines + Sulfonamides	Trimethoprim	*Salmonella* spp., *E. coli*, *Enterococcus* spp., *Staphylococcus* spp.
	Sulfamethaxazole	*Salmonella* spp., *E. coli*, *L. monocytogenes*, *Staphylococcus* spp.
	Sulfadiazine	*Salmonella* spp.
Fluoroquinolones	Enrofloxacin	*E. coli*, *Campylobacter* spp.
	Ciprofloxacin	*Salmonella* spp., *E. coli*, *Campylobacter* spp., *L. monocytogenes*, *Enterococcus* spp., *Staphylococcus* spp.
	Levofloxacin	*Salmonella* spp., *Campylobacter* spp.
	Moxifloxacin	*L. monocytogenes*
	Marbofloxacin	*Salmonella* spp.

*E. coli*=*Escherichia coli*, *L. monocytogenes*=*Listeria monocytogenes*
